# Impact of phthalate and BPA exposure during in utero windows of susceptibility on reproductive hormones and sexual maturation in peripubertal males

**DOI:** 10.1186/s12940-017-0278-5

**Published:** 2017-06-21

**Authors:** Deborah J. Watkins, Brisa N. Sánchez, Martha Maria Téllez-Rojo, Joyce M. Lee, Adriana Mercado-García, Clara Blank-Goldenberg, Karen E. Peterson, John D. Meeker

**Affiliations:** 1 Department of Environmental Health Sciences, University of Michigan School of Public Health, 6611C SPH I, 1415 Washington Heights, Ann Arbor, MI 48109 USA; 2 Department of Biostatistics, University of Michigan School of Public Health, Ann Arbor, MI USA; 3 Center for Nutrition and Health Research, Instituto Nacional de Salud Pública, MOR, Cuernavaca, Mexico; 4 Pediatric Endocrinology, Child Health Evaluation and Research Unit (CHEAR), University of Michigan, Ann Arbor, MI USA; 5 American British Cowdray (ABC) Hospital, Mexico City, Mexico; 6 Department of Nutritional Sciences, University of Michigan School of Public Health, Ann Arbor, MI USA; 7 Center for Human Growth and Development, University of Michigan, Ann Arbor, MI USA

**Keywords:** In utero exposure, Windows of susceptibility, Puberty, Phthalates, BPA

## Abstract

**Background:**

Phthalates and BPA are endocrine disrupting chemicals (EDCs) widely used in consumer products. Evidence suggests that phthalate and BPA exposure alters steroid hormone levels in adults, while in utero exposure has been associated with altered fetal reproductive development in boys. However, the impact of exposure during distinct critical windows of in utero development on hormone concentrations and sexual maturation during the pubertal transition has not been examined. The objective of this study was to assess trimester-specific in utero phthalate and BPA exposure in relation to measures of reproductive development among peripubertal boys in a Mexico City birth cohort.

**Methods:**

We measured maternal urinary phthalate metabolites and BPA during the first, second, and third trimesters of pregnancy. We measured serum levels of testosterone, estradiol, dehydroepiandrosterone sulfate (DHEA-S), inhibin B, and sex hormone-binding globulin (SHBG), and assessed sexual maturation (Tanner staging and testicular volume) among male children at age 8–14 years (*n* = 109). Linear and logistic regression were used to investigate trimester-specific in utero exposure as predictors of peripubertal hormone levels and sexual maturation, respectively. In sensitivity analyses we evaluated estimated exposure at 7 weeks gestation and rates of change in exposure across pregnancy in relation to outcomes.

**Results:**

Exposure to phthalates during the third trimester was associated with reduced odds of having a Tanner stage >1 for pubic hair development (e.g. MBzP OR = 0.18 per interquartile range (IQR) increase; 95% CI:0.03–0.97) and higher peripubertal SHBG levels (e.g. MBzP 15.2%/IQR; 95% CI:3.2–28%), while first and second trimester phthalates were not. In contrast, exposure to DEHP during the first trimester was associated with higher estradiol (11%/IQR; 95% CI:1.5–22%), while second or third trimester DEHP exposure was not. Sensitivity analyses yielded similar findings.

**Conclusions:**

Associations between in utero phthalate and BPA exposure and peripubertal measures of male reproductive development are dependent on the timing of that exposure during gestation. These findings suggest that future epidemiological studies relating in utero EDC exposure to pubertal outcomes should consider windows of susceptibility.

**Electronic supplementary material:**

The online version of this article (doi:10.1186/s12940-017-0278-5) contains supplementary material, which is available to authorized users.

## Background

Phthalates and bisphenol A (BPA) are endocrine disrupting chemicals (EDCs) [1] that are widely used in a range of consumer products, resulting in ubiquitous human exposure [2–4]. Evidence suggests that exposure to phthalates and BPA alters reproductive hormone levels in adults [5–9], while exposure during in utero development has been associated with changes in reproductive hormone levels in newborns [10, 11]. In addition, several studies have reported associations between in utero phthalate exposure and shortened anogenital distance (AGD) [12–14], a marker of decreased androgen exposure during in utero reproductive development and a potential indicator of reduced male fertility in adulthood [15, 16]. However, the impact of in utero phthalate and BPA exposure on peripubertal reproductive hormone concentrations and sexual maturation is unclear.

In earlier work, we reported that phthalate exposure during the third trimester of in utero development was associated with lower odds of adrenarche, while peripubertal exposure was not [17]. Given that androgen action during relatively short, specific fetal periods is necessary for male reproductive development [18, 19], the objective of the present analysis was to utilize newly available data to assess early in utero windows of susceptibility to phthalate and BPA exposure among boys in this study population. To our knowledge, this is the first study to evaluate maternal urinary phthalate metabolite and BPA concentrations during their first, second, and third trimesters of pregnancy, as well as the level and rate of change of exposure across pregnancy, in relation to peripubertal reproductive hormone levels and measures of sexual maturation among their male children.

## Methods

### Study population

Participants are part of the Early Life Exposure in Mexico to Environmental Toxicants (ELEMENT) project, a longitudinal cohort study of pregnant women in Mexico City and their offspring. Our analysis includes women who were recruited from maternity hospitals during their first trimester between 1997 and 2004 and their offspring as previously described [20]. Mothers provided a urine sample and completed interview-based questionnaires at up to three different prenatal visits (mean gestational age at visit 1: 13.5 weeks [range 5–24], visit 2: 25.1 weeks [range 17–37], and visit 3: 34.4 weeks [range 26–43]). Gestational age was based on the self-reported date of each mother’s last menstrual period. In 2010, a subset of their children, who were then 8 to 14 years of age, were re-contacted to participate in follow-up studies (*n* = 250). Participants were selected from the overall ELEMENT study based on the availability of archived prenatal urine samples for biomarker measurement. Children provided serum samples, anthropometry, and demographic information via an interview-based questionnaire. Age-specific body mass index (BMI) z-scores were calculated based on the World Health Organization growth standards for age and sex [21]. In the current analyses, we included male children for whom we had maternal urinary phthalate metabolite and BPA measurements from at least one prenatal study visit (*n* = 109). Research protocols were approved by the ethics and research committees of the Mexico National Institute of Public Health and the University of Michigan, and all participants provided informed consent prior to enrollment.

### Urinary phthalate metabolites and BPA

Each mother provided a second morning void urine sample during at least one of the three prenatal study visits (visit 1 *n* = 92, visit 2 *n* = 91, visit 3 *n* = 108; *n* = 85 provided a sample at all three time points). Samples were frozen and stored at −80 °C at the University of Michigan until analysis at NSF International (Ann Arbor, MI). BPA and nine phthalate metabolites, including monoethyl phthalate (MEP), mono-*n*-butyl phthalate (MnBP), monoisobutyl phthalate (MiBP), monobenzyl phthalate (MBzP), mono-3-carboxypropyl phthalate (MCPP), mono-2-ethylhexyl phthalate (MEHP), mono-2-ethyl-5-hydroxyhexyl phthalate (MEHHP), mono-2-ethyl-5-oxohexyl phthalate (MEOHP), and mono-2-ethyl-5-carboxypentyl phthalate (MECPP) were measured using isotope dilution–liquid chromatography–tandem mass spectrometry (ID–LC–MS/MS) as previously described [20]. We calculated a di-2-ethylhexyl phthalate (DEHP) metabolite summary measure (ΣDEHP) for each sample by dividing individual MEHP, MEHHP, MEOHP, and MECPP concentrations by their molar mass and summing them. We calculated a summary dibutyl phthalate (DBP) metabolite measure (ΣDBP) as the molar sum of MnBP and MiBP. Specific gravity (SG) was measured using a handheld digital refractometer (Atago Co., Ltd., Tokyo, Japan) at the time of sample analysis. For select analyses only, phthalate metabolite and BPA values were corrected for urinary SG using the following equation: P_c_ = P[(SG_p_ – 1)/(SG_i_ – 1)] where P_c_ is the SG-corrected phthalate metabolite or BPA concentration (ng/mL), P is the measured phthalate metabolite or BPA concentration, SG_p_ is the median urinary specific gravity, and SG_i_ is the individual’s urinary specific gravity. Spearman correlations between trimester specific measures of SG, phthalate metabolites, and BPA are presented in (Additional file 1: Table S1). Values below the limit of detection (LOD) were replaced with the LOD/√2.

### Hormones

Children provided fasting blood samples during the follow-up visit at age 8–14 years. Serum aliquots were separated and frozen at −80 °C, and then sent to the Clinical Ligand Assay Service Satellite (CLASS) Laboratory at the University of Michigan (Ann Arbor, MI) for hormone analysis. We measured estradiol, testosterone, inhibin B, and sex hormone-binding globulin (SHBG) as biomarkers of puberty, and dehydroepiandrosterone sulfate (DHEA-S) as a biomarker of adrenarche. Estradiol, total testosterone, SHBG, and DHEA-S were measured using an automated chemiluminescent immunoassay (Bayer Diagnostics ACS:180). Active inhibin B was assayed using Gen II ELISA (Beckman Coulter, Webster, TX). Values below the limit of detection (LOD) were replaced with the LOD/√2.

### Sexual maturation

A trained pediatrician evaluated male offspring for Tanner staging and testicular volume using standardized protocols. We used genital stage (GD) as an indicator of puberty and pubic hair stage (PH) as an indicator of adrenarche, with stage 1 corresponding to no development and stage 5 corresponding to full development [22]. For analyses, Tanner stages were converted to dichotomous variables with pubertal onset defined by having a GD stage >1 and adrenarche defined by having a PH stage >1. Right and left testicular volume (TV) were measured with an orchidometer, and the larger of the two measurements was used in analyses. As a testicular volume of 1–3 mL is considered prepubertal, a cutoff of 3 mL was used to denote no puberty (≤3 mL) vs. puberty (>3 mL) [23].

### Statistical methods

Serum hormone and urinary phthalate metabolite and BPA concentrations were log-normally distributed, and ln-transformed prior to analysis. We used mixed models to calculate intraclass correlation coefficients (ICCs) and 95% confidence intervals to examine the variability of phthalate and BPA measurements across pregnancy within individuals with more than one exposure measurement [24]. Linear regression was used to separately assess associations between visit-specific phthalate metabolite and BPA concentrations and peripubertal serum hormone concentrations, adjusting a priori for child age and BMI Z-score, and for SG as a measure of urinary dilution. To assess overall exposure during pregnancy, we calculated geometric mean (GM) phthalate metabolite and BPA concentrations for each individual using all available measurements from prenatal visits 1, 2, and 3. If only one measurement from one prenatal visit was available, that value was used as the GM. Individual GM values were then entered into regression models to assess relationships between overall in utero exposure and peripubertal hormone levels. Results are presented as the percent change in hormone (95% confidence interval) per interquartile range (IQR) increase in phthalate metabolite or BPA. All results are calculated based on the IQR of GM concentrations across pregnancy in order to more easily compare visit-specific effect estimates.

We used logistic regression to separately examine associations between visit-specific urinary phthalate metabolite or BPA concentrations and the odds of pubertal onset or adrenarche using dichotomous measures of sexual maturation (GD, PH, TV). In full models, child age and BMI z-score were included as covariates a priori. As few participants were categorized as having a PH Tanner stage >1 (Additional file 1: Table S2), the number of covariates we could reliably enter into models was limited [25, 26]. Thus, to minimize the number of covariates in logistic models, we included SG-corrected phthalate metabolite and BPA concentrations rather than entering SG as a separate covariate. Individual GM phthalate metabolite and BPA values were also entered into logistic regression models to assess relationships of overall in utero exposure with odds of pubertal onset and adrenarche. Results are presented as odds ratios (OR, 95% confidence interval), which express the change in odds per IQR increase in exposure, with the IQR based on GM concentrations across pregnancy.

We carried out complementary, sensitivity analyses for each exposure that utilized data from all participants and all visits in a single model to examine whether the level (intercept) or rate of change (slope) of the exposure during pregnancy was associated with hormone concentrations or sexual maturation. To carry out this analysis, we first used mixed effects models with gestational age as a predictor of ln-transformed, SG-corrected phthalate or BPA concentrations to estimate, for each woman and each compound, a random intercept and slope that described the compound’s trajectory during the course of pregnancy. Prior to entering gestational age into the model, it was centered at 7 weeks, such that an individual’s intercept represents urinary phthalate metabolite or BPA concentrations at 7 weeks, the mid-point of the first trimester of pregnancy. An individual’s slope indicates the rate of change in urinary phthalate metabolite or BPA across pregnancy. For each exposure, we then entered the estimated slope and intercept variables into regression models as predictors of either hormone concentrations or measures of sexual maturation. However, if the slope and intercept for a specific phthalate metabolite or BPA were highly correlated (Pearson *r* > 0.8), or if effect estimates were imprecise (e.g. infinitely wide 95% confidence intervals), only the intercept was used. Findings were considered statistically significant at *p* < 0.05, and all analyses were performed using SAS version 9.3 (Cary, NC).

## Results

The percent of samples with phthalate metabolite or BPA concentrations below the limit of detection, as well as the geometric means and standard deviations of sample concentrations from prenatal visits 1, 2, and 3 are shown in Table 1. Urinary BPA concentrations significantly decreased, while MBzP, MEOHP, and MiBP concentrations significantly increased across pregnancy. ICCs for phthalate metabolite and BPA concentrations among all mothers ranged from 0.21 for MBzP to 0.41 for MEP, suggesting substantial within individual variability. Differences in levels across pregnancy and the percent of samples below the LOD were similar when we limited our analysis to only mothers who had male children, although ICCs ranged from 0.14 for ΣDEHP to 0.47 for MEP. The distribution of serum hormone levels and measures of sexual maturation among this population have been previously reported elsewhere [17].

**Table 1:** Distribution of urinary phthalate metabolites and BPA across pregnancy (μg/L)

		Visit 1 (*n* = 199)			Visit 2 (*n* = 200)			Visit 3 (*n* = 225)			Prenatal GM (*n* = 229)			Variability		
All	% < LOD^a^	GM (GSD)		Max	GM (GSD)		Max	GM (GSD)		Max	GM (GSD)		Max	ICC	(95% CI)	
BPA^b^	23.6	1.0	(2.5)	9.3	1.0	(2.4)	13.2	0.7	(2.30	18.7	0.89	(2.0	6.9	0.32	(0.24,	0.41)
MEHP	8.0	4.9	(2.8)	125	4.7	(2.9)	152	5.0	(2.8)	215	5.0	(2.2)	215	0.25	(0.17,	0.35)
MEHHP	0	16.6	(3.1)	158	17.4	(3.2)	1290	19.6	(2.9)	383	18.6	(2.3)	383	0.23	(0.15,	0.33)
MEOHP^b^	0	8.9	(3.1)	85.0	10.4	(3.2)	730	11.8	(2.9)	244	10.8	(2.3)	244	0.23	(0.15,	0.33)
MECPP	0.2	30.0	(2.7)	252	31.9	(2.7)	2650	31.9	(2.7)	414	32.3	(2.1)	414	0.26	(0.18,	0.36)
MBzP^b^	3.2	2.6	(4.0)	69.6	2.4	(3.6)	38.5	4.2	(2.6)	109	3.2	(2.4)	34.5	0.21	(0.14,	0.31)
MBP	0.3	59.0	(4.1)	1340	49.0	(3.8)	1390	54.5	(3.4)	1190	56.0	(2.6)	468	0.28	(0.20,	0.38)
MiBP^b^	22.0	0.92	(4.7)	118	0.7	(4.6)	26.5	1.9	(2.9)	40.1	1.2	(2.9)	32.4	0.31	(0.23,	0.40)
MCPP	8.0	1.1	(2.9)	17.8	1.1	(2.8)	15.0	1.1	(2.7)	12.1	1.1	(2.2)	6.6	0.31	(0.23,	0.40)
MEP	0.3	138	(4.2)	4480	119	(3.9)	4980	112	(4.2)	9810	126	(3.1)	2285	0.41	(0.33,	0.50)
ΣDEHP^c^		0.21	(2.7)	1.7	0.22	(2.9)	16.0	0.24	(2.7)	4.3	0.23	(2.1)	4.3	0.24	(0.16,	0.34)
ΣDBP^d^		0.27	(4.1)	6.1	0.23	(3.7)	6.3	0.26	(3.3)	5.4	0.26	(2.6)	2.2	0.28	(0.20,	0.38)
		Visit 1 (*n* = 92)			Visit 2 (*n* = 91)			Visit 3 (*n* = 108)			Prenatal GM (*n* = 109)			Variability		
Males	% < LOD	GM (GSD)		Max	GM (GSD)		Max	GM (GSD)		Max	GM (GSD)		Max	ICC	(95% CI)	
BPA^b^	26.8	0.93	(2.5)	7.9	0.87	(2.3)	9.2	0.64	(2.3)	9.0	0.81	(1.9)	4.5	0.28	(0.17,	0.42)
MEHP	7.9	4.8	(2.6)	53.0	4.4	(2.8)	50.3	5.0	(2.9)	215	5.0	(2.2)	215	0.28	(0.17,	0.43)
MEHHP	0	15.6	(3.4)	150	15.7	(3.0)	85.0	19.9	(2.9)	383	18.0	(2.3)	383	0.15	(0.06,	0.33)
MEOHP^b^	0	8.3	(3.3)	80.2	9.4	(3.0)	53.5	12.0	(2.8)	244	10.4	(2.3)	244	0.15	(0.06,	0.33)
MECPP	0.3	28.5	(2.9)	227	29.4	(2.5)	134	32.7	(2.5)	414	31.5	(2.1)	414	0.15	(0.05,	0.33)
MBzP^b^	3.4	2.4	(4.3)	51.3	2.5	(3.8)	34.5	4.3	(2.5)	32.5	3.3	(2.5)	34.5	0.15	(0.06,	0.33)
MBP	0.7	54.1	(4.2)	870	42.3	(3.7)	655	53.9	(3.1)	1000	53.0	(2.6)	468	0.24	(0.14,	0.40)
MiBP^b^	25.1	0.76	(4.7)	118	0.62	(4.5)	25.8	1.7	(2.8)	40.1	1.1	(2.9)	32.4	0.32	(0.21,	0.46)
MCPP	7.2	1.1	(2.7)	9.8	1.1	(2.7)	8.2	1.1	(2.6)	12.1	1.1	(2.1)	6.0	0.27	(0.16,	0.41)
MEP	0.3	128	(4.2)	3260	117	(3.6)	2160	110	(3.7)	7950	124	(3.0)	2285	0.47	(0.36,	0.60)
ΣDEHP^c^		0.20	(2.9)	1.6	0.20	(2.7)	0.93	0.24	(2.6)	4.3	0.22	(2.1)	4.3	0.14	(0.05,	0.32)
ΣDBP^d^		0.25	(4.1)	4.5	0.20	(3.7)	3.0	0.25	(3.1)	4.5	0.25	(2.6)	2.1	0.25	(0.15,	0.40)

*LOD* limit of detection, *GM* geometric mean of samples collected during pregnancy, *GSD* geometric standard deviation, *ICC* intraclass correlation coefficient, *CI* confidence interval

^a^LODs: BPA = 0.4, MEHP, MEP = 1.0, MEHHP, MEOHP = 0.1, MECPP, MBzP, MiBP, MCPP = 0.2, MBP = 0.5; ^b^Concentrations were significantly different across prenatal visits (*p* < 0.05); ^c^ΣDEHP = molecular sum of MEHP, MEHHP, MEOHP, and MECPP (μmol/L); ^d^ΣDBP = molar sum of MnBP and MiBP (μmol/L)

Maternal phthalate metabolite and BPA concentrations at prenatal visit 1 were not associated with peripubertal hormone levels in male offspring at 8–14 years of age, with the exception of ΣDEHP, for which an IQR increase was associated with 11% (95% CI: 1.5, 21.9) higher serum estradiol after adjustment for child age, BMI z-score, and urinary SG (Table 2). At prenatal visit 2, an IQR increase in MEP was associated with 15% (95% CI: −25.1, −3.6) lower estradiol concentrations, while an IQR increase in MBzP was associated with 16% (95% CI: −26.8, −3.4) lower DHEA-S. Maternal BPA concentrations at prenatal visit 2 were also associated with higher inhibin B levels, and MBzP, MCPP, and ΣDBP at prenatal visit 3 were all associated with higher SHBG levels. Geometric mean levels of phthalate metabolite and BPA concentrations across pregnancy were not significantly associated with peripubertal serum hormone concentrations (Table 2).

**Table 2:** Percent difference in peripubertal hormone levels associated with an IQR increase in phthalate metabolite or BPA concentration during trimester-specific in utero development^a^

	Visit 1 (*n* = 91)	Visit 2 (*n* = 90)	Visit 3 (*n* = 107)	Prenatal GM (*n* = 108)
	%Δ/IQR (95% CI)	%Δ/IQR (95% CI)	%Δ/IQR (95% CI)	%Δ/IQR (95% CI)
Estradiol				
BPA	−1.1 (−10.8, 9.7)	−4.5 (−14.2, 6.2)	0.1 (−9.1, 10.2)	−2.4 (−13.7, 10.4)
MBzP	5.0 (−3.5, 14.3)	0.7 (−9.2, 11.8)	−2.0 (−11.7, 8.8)	3.6 (−7.4, 16.0)
MCPP	5.3 (−6.2, 18.3)	3.4 (−9.8, 18.6)	3.7 (−5.7, 14.1)	6.2 (−7.4, 21.8)
MEP	3.0 (−7.9, 15.2)	−15.0 (−25.1, −3.6)*	−0.6 (−10.2, 10.2)	−3.4 (−14.6, 9.4)
ΣDEHP	11.2 (1.5, 21.9)*	8.2 (−3.2, 21.0)	4.6 (−4.8, 14.9)	11.2 (−1.4, 25.3)
ΣDBP	8.5 (−0.7, 18.5)	−5.8 (−15.4, 4.8)	7.6 (−2.6, 18.7)	6.5 (−5.5, 20.1)
Testosterone				
BPA	9.0 (−16.6, 42.3)	14.8 (−12.3, 50.4)	10.1 (−16.7, 45.5)	33.4 (−6.1, 89.4)
MBzP	17.1 (−5.9, 45.7)	−2.5 (−25.1, 27.0)	3.1 (−23.9, 39.7)	14.6 (−17.1, 58.5)
MCPP	9.9 (−18.8, 48.6)	14.8 (−18.8, 62.4)	9.5 (−17.1, 44.6)	27.2 (−14.2, 88.7)
MEP	30.3 (−2.0, 73.2)	−2.8 (−30.3, 35.6)	−8.2 (−31.8, 23.5)	6.6 (−25.4, 52.5)
ΣDEHP	10.8 (−13.1, 41.4)	2.5 (−23.1, 36.5)	8.4 (−17.6, 42.7)	20.1 (−15.2, 70.1)
ΣDBP	4.8 (−17.0, 32.4)	−2.9 (−26.1, 27.7)	−9.3 (−32.1, 21.2)	−0.3 (−29.6, 41.2)
SHBG				
BPA	−0.8 (−11.1, 10.6)	4.7 (−6.2, 16.9)	5.3 (−5.0, 16.8)	5.6 (−7.4, 20.5)
MBzP	5.1 (−3.9, 14.9)	−1.2 (−11.3, 10.0)	15.2 (3.2, 28.5)*	10.2 (−2.2, 24.2)
MCPP	4.6 (−7.4, 18.3)	−2.3 (−15.2, 12.5)	14.4 (3.5, 26.5)*	12.5 (−2.8, 30.2)
MEP	3.4 (−8.1, 16.3)	−2.8 (−15.0, 11.3)	4.6 (−6.4, 16.8)	4.2 (−8.8, 19.0)
ΣDEHP	−0.2 (−9.7, 10.2)	1.9 (−9.3, 14.4)	8.4 (−2.1, 19.9)	8.2 (−4.9, 23.1)
ΣDBP	1.1 (−8.0, 11.2)	−6.5 (−16.2, 4.4)	12.3 (1.1, 24.9)*	5.2 (−7.6, 19.7)
DHEA-S				
BPA	3.5 (−10.8, 20.0)	−12.1 (−23.9, 1.6)	1.2 (−12.4, 16.9)	−1.9 (−18.4, 18.0)
MBzP	−0.9 (−12.3, 12.1)	−15.9 (−26.8, −3.4)*	−4.4 (−18.3, 11.8)	−11.9 (−25.5, 4.0)
MCPP	−2.3 (−17.4, 15.5)	−7.4 (−23.3, 11.8)	−5.0 (−17.6, 9.7)	−6.0 (−23.5, 15.4)
MEP	−4.9 (−19.0, 11.7)	−9.2 (−24.1, 8.6)	−7.7 (−20.8, 7.5)	−9.4 (−24.7, 8.9)
ΣDEHP	0.9 (−11.9, 15.5)	−4.4 (−18.2, 11.7)	−0.1 (−13.3, 15.1)	0.5 (−16.2, 20.5)
ΣDBP	−1.1 (−13.1, 12.6)	−7.3 (−20.0, 7.5)	−13.5 (−25.3, 0.2)	−10.8 (−25.4, 6.7)
Inhibin B				
BPA	3.0 (−7.7, 14.8)	11.8 (0.4, 24.6)*	−1.8 (−12.0, 9.6)	8.8 (−5.2, 24.9)
MBzP	2.7 (−6.1, 12.4)	−2.9 (−12.8, 8.1)	−6.5 (−17.0, 5.3)	−5.6 (−16.8, 7.2)
MCPP	4.2 (−7.8, 17.8)	8.4 (−5.9, 24.7)	−0.9 (−11.2, 10.6)	4.9 (−10.2, 22.5)
MEP	−1.6 (−12.6, 10.7)	−1.3 (−13.9, 13.0)	−13.6 (−22.9, −3.2)*	−8.8 (−20.6, 4.9)
ΣDEHP	8.1 (−2.0, 19.3)	7.0 (−4.7, 20.2)	−2.9 (−12.8, 8.2)	5.1 (−8.3, 20.5)
ΣDBP	2.9 (−6.4, 13.2)	8.3 (−3.0, 21.0)	−4.1 (−14.4, 7.5)	2.3 (−10.7, 17.2)

*p < 0.05

^a^Adjusted for child age, BMI z-score, and urinary specific gravity

Although logistic regression models including SG as a separate covariate (Additional file 1: Table S3) yielded results similar to models including SG-corrected phthalate metabolite and BPA values, the latter generally provided narrower confidence intervals. Therefore, we have presented ORs from models with SG-corrected exposure measures in Table 3. With the exception of MEP at visit 1, maternal phthalate metabolite and BPA levels at prenatal visits 1 and 3, as well as the GM of concentrations across pregnancy, were all associated with reduced odds of adrenarche (PH >1) after adjustment for child age and BMI z-score (Table 3). However, MBzP and ΣDEHP at visit 3, and the GM of MBzP across pregnancy were the only associations to reach statistical significance. Pubertal onset (GD >1 or TV >3 mL) was not associated with prenatal phthalate metabolite and BPA concentrations measured at any study visit or with GM concentrations across pregnancy. The one exception to this was reduced odds of TV > 3 mL associated with higher GM MBzP concentrations.

**Table 3:** Odds of Tanner stage >1 or TV >3 ml associated with an IQR increase in trimester-specific, SG-corrected in utero urinary phthalate metabolite or BPA concentration^a^

	Visit 1 (*n* = 90)	Visit 2 (*n* = 89)	Visit 3 (*n* = 105)	Prenatal GM (*n* = 106)
	OR/IQR (95% CI)	OR/IQR (95% CI)	OR/IQR (95% CI)	OR/IQR (95% CI)
Genital Development				
BPA	0.71 (0.39, 1.28)	1.41 (0.78, 2.54)	0.93 (0.53, 1.61)	1.04 (0.5, 2.14)
MBzP	0.95 (0.58, 1.55)	0.98 (0.57, 1.68)	0.71 (0.37, 1.36)	0.75 (0.37, 1.51)
MCPP	1.2 (0.61, 2.36)	1.16 (0.53, 2.54)	0.97 (0.53, 1.77)	1.14 (0.48, 2.73)
MEP	1.5 (0.79, 2.85)	1.17 (0.56, 2.48)	1.09 (0.58, 2.03)	1.27 (0.58, 2.81)
ΣDEHP	0.99 (0.56, 1.74)	0.85 (0.46, 1.57)	0.71 (0.4, 1.29)	0.77 (0.37, 1.62)
ΣDBP	0.77 (0.47, 1.27)	0.86 (0.48, 1.52)	0.59 (0.31, 1.13)	0.57 (0.27, 1.21)
Pubic Hair				
BPA	0.2 (0.02, 2.76)	1.1 (0.31, 3.9)	0.42 (0.13, 1.36)	0.72 (0.19, 2.73)
MBzP	0.13 (0.01, 1.56)	0.99 (0.26, 3.73)	0.18 (0.03, 0.97)*	0.31 (0.1, 0.93)*
MCPP	0.33 (0.02, 4.72)	5.99 (0.5, 72.02)	0.47 (0.16, 1.37)	0.5 (0.11, 2.26)
MEP	2.04 (0.36, 11.58)	1.31 (0.22, 7.85)	0.44 (0.15, 1.24)	0.52 (0.17, 1.6)
ΣDEHP	0 (0, 542.1)	2.22 (0.28, 17.34)	0.27 (0.07, 0.99)*	0.16 (0.03, 1.02)
ΣDBP	0.28 (0.02, 3.85)	2.68 (0.48, 15.08)	0.36 (0.13, 1.06)	0.39 (0.11, 1.42)
Testicular Volume				
BPA	1.28 (0.67, 2.45)	2.01 (0.94, 4.28)	1.34 (0.68, 2.66)	2.29 (0.87, 5.98)
MBzP	0.7 (0.39, 1.23)	0.62 (0.3, 1.29)	0.73 (0.33, 1.6)	0.4 (0.16, 1)*
MCPP	0.56 (0.25, 1.27)	1.59 (0.6, 4.23)	1.03 (0.5, 2.12)	1.07 (0.36, 3.19)
MEP	1.33 (0.64, 2.76)	0.75 (0.31, 1.86)	1.32 (0.6, 2.91)	1.16 (0.44, 3.06)
ΣDEHP	1.21 (0.64, 2.3)	0.99 (0.49, 2)	1.5 (0.75, 3)	1.94 (0.7, 5.34)
ΣDBP	1 (0.54, 1.84)	1.66 (0.85, 3.25)	0.88 (0.44, 1.75)	1.35 (0.54, 3.4)

*p < 0.05

^a^Adjusted for child age and BMI z-score

Population means (fixed effects) and distributions of individual exposure intercepts and slopes (random effects) from mixed models evaluating the effects of gestational age on prenatal phthalate and BPA levels are presented in Additional file 1: Table S4. When we considered individual’s exposure intercept and slope as predictors of peripubertal hormone levels we observed relationships that were consistent with what we observed using the visit specific concentrations. For instance, an IQR increase in the ΣDEHP intercept, which represents exposure in the first trimester, was associated with 15% higher peripubertal estradiol concentrations (Table 4). This is consistent with our main analyses, in which we observed 11% higher estradiol with an IQR increase ΣDEHP at visit 1 (Table 2). Further, an IQR increase in MEP slope, which represents the change in exposure across pregnancy, was associated with 13% higher inhibin B (Table 4), while in our main analysis an IQR increase in MEP levels at prenatal visit 3 was significantly associated with 14% higher inhibin B (Table 2). Consistent with results from our main analysis in Table 3, estimated exposure at 7 weeks (exposure intercepts) were not significantly associated with odds of puberty or adrenarche (GD >1, TV >3, or PH >1) (Table 5), although estimated relationships were in similar directions.

**Table 4:** Relationships between IQR increases in estimated intercepts and slopes of exposure across in utero development and hormone levels at 8–14 years (*n* = 104)^a^

		Estradiol	Testosterone	SHBG	DHEA-S	Inhibin B
		%Δ/IQR (95% CI)	%Δ/IQR (95% CI)	%Δ/IQR (95% CI)	%Δ/IQR (95% CI)	%Δ/IQR (95% CI)
BPA^b^	Intercept	−3.6 (−13.6, 7.5)	12.8 (−16, 51.6)	2.3 (−8.8, 14.9)	−0.9 (−15.1, 15.7)	5.7 (−6.1, 18.9)
	Slope	n/a	n/a	n/a	n/a	n/a
MBzP^b^	Intercept	6.1 (−4.5, 17.9)	20.4 (−9.5, 60.1)	9.8 (−1.7, 22.6)	−5.4 (−18.5, 9.9)	1.5 (−9.6, 13.8)
	Slope	n/a	n/a	n/a	n/a	n/a
MCPP	Intercept	8.3 (−5.3, 24.0)	9.4 (−24.2, 57.8)	15.3 (0.3, 32.6)*	−7.1 (−23.2, 12.5)	5.6 (−8.7, 22.3)
	Slope	2.7 (−9.7, 16.9)	−0.5 (−30.0, 41.3)	14.5 (0.2, 30.9)*	−7.3 (−22.8, 11.4)	−1.4 (−14.3, 13.3)
MEP	Intercept	−6.3 (−17.2, 6.1)	6.7 (−23.5, 48.8)	4.9 (−8.1, 19.7)	−6.7 (−21.8, 11.3)	−6.8 (−18.3, 6.4)
	Slope	−7.4 (−16.8, 3.1)	−22.1 (−41.6, 3.8)	0.0 (−10.8, 12.1)	−4.8 (−18.3, 10.9)	−12.9 (−22.3, −2.4)*
ΣDEHP	Intercept	15.0 (3.2, 28.2)*	6.2 (−21.6, 43.9)	9.4 (−2.8, 23.0)	0.4 (−14.4, 17.7)	6.6 (−5.4, 20.2)
	Slope	0.9 (−9.7, 12.8)	−9.1 (−33.3, 24.0)	9.2 (−3.2, 23.0)	−2.2 (−16.8, 15.1)	−6.6 (−17.3, 5.6)
ΣDBP	Intercept	8.7 (−7.0, 27.1)	−12.4 (−42.6, 33.7)	11.2 (−5.7, 31.1)	−14.9 (−31.7, 5.9)	5.1 (−11.3, 24.5)
	Slope	0.7 (−12.1, 15.2)	−19.8 (−44.4, 15.7)	10.5 (−4.2, 27.4)	−14.4 (−29.2, 3.6)	−2.7 (−15.9, 12.7)

*p < 0.05

^a^Adjusted for child age and BMI z-score. ^b^Intercept and slope highly correlated (BPA *r* = −0.82, MBzP *r* = −0.99), only intercept included in model

**Table 5:** Relationships between an IQR increase in estimated exposure at 7 weeks gestation (intercepts) and odds of Tanner stage >1 or TV >3 mL (*n* = 102)^a^

	Genital Development	Pubic Hair	Testicular Volume
	OR/IQR (95% CI)	OR/IQR (95% CI)	OR/IQR (95% CI)
Intercept			
BPA	0.86 (0.46, 1.58)	0.76 (0.20, 2.98)	1.67 (0.79, 3.56)
MBzP	0.82 (0.44, 1.52)	0.54 (0.18, 1.62)	0.55 (0.25, 1.22)
MCPP	1.17 (0.64, 2.15)	1.33 (0.43, 4.15)	0.66 (0.31, 1.43)
MEP	1.33 (0.67, 2.61)	1.08 (0.33, 3.54)	1.26 (0.56, 2.86)
ΣDEHP	0.82 (0.46, 1.45)	0.41 (<0.001, 1.46)	1.29 (0.65, 2.56)
ΣDBP	0.65 (0.34, 1.23)	0.94 (0.29, 3.09)	1.29 (0.56, 2.96)

^a^Adjusted for child age and BMI z-score

## Discussion

We investigated maternal urinary phthalate metabolite and BPA concentrations during the first, second, and third trimesters of pregnancy in relation to peripubertal reproductive hormone levels and measures of sexual maturation in male offspring in order to identify potential windows of exposure susceptibility during in utero development. Our findings suggest that compared to phthalate exposure early in pregnancy, exposure specifically during the third trimester is associated with reduced odds of adrenarche and a corresponding increase in serum SHBG concentrations. In addition, we observed an increase in peripubertal serum estradiol associated with DEHP exposure specifically in the first trimester of in utero development.

In comparison with our previous evaluation of third trimester phthalate levels and sexual maturation in males in this same population [17], estimated associations for first and second trimester exposure also indicated reduced odds of adrenarche (PH >1) with phthalate exposure, although associations did not reach statistical significance. Consistent with reduced odds of adrenarche, we observed reduced peripubertal serum DHEA-S concentrations in relation to several phthalate metabolite levels across pregnancy, although only MBzP in the second trimester was statistically significant (Table 2).

Two prior studies examined associations between markers of in utero phthalate exposure and reproductive hormone concentrations in cord blood, one of which reported no relationship between third trimester maternal urinary phthalate levels and hormones among male newborns [27]. However, the second study reported associations between third trimester MEHP levels in maternal blood and reduced testosterone to estradiol ratios and inhibin B among males [10]. In addition, a cross-sectional study of perinatal phthalate exposure reported that MEP and MBP concentrations in breast milk were associated with increased luteinizing hormone to free testosterone ratios and SHBG in male infants at three months of age [28]. Findings from the latter two studies, as well as the associations between several phthalate metabolite levels during the third trimester and higher peripubertal SHBG concentrations reported in the present study, are consistent with anti-androgen exposure.

A number of studies have examined associations between in utero phthalate exposure and fetal reproductive tract development in males. One of the first studies of phthalate exposure found that maternal urinary MEP, MBP, MBzP, and MiBP levels during the third trimester were associated with shortened AGD in male infants [12]. In a recent study, researchers found that maternal urinary DEHP metabolite concentrations in the first trimester, but not the second and third trimesters, were inversely associated with this same outcome measure [13]. This is consistent with what is currently known about male reproductive development, as AGD is determined by androgen action during the masculinizing programming window, which in humans is thought to be between weeks 7–15 of in utero development [19]. Interestingly, we observed a similar relationship between DEHP metabolites in the first trimester and increased serum estradiol levels in males at 8–14 years of age, but saw no association with exposure during later trimesters. However, we did not observe associations between in utero phthalate exposure and peripubertal testosterone levels, as we might expect given phthalates anti-androgenic activity.

One previous study reported positive correlations between cord blood levels of BPA with inhibin B and testosterone among male infants [11], which although measured in different media and at different time points, is consistent with our finding of increased peripubertal inhibin B concentrations in relation to urinary BPA at prenatal visit 2. However, in the current study, prenatal BPA levels were not associated with any other hormone or measure of sexual maturation among boys at 8–14 years of age.

Although mechanisms by which in utero phthalate exposure may alter fetal and pubertal reproductive development are unclear, animal studies have shown that prenatal phthalate exposure may interfere with differentiation and function of both Sertoli and Leydig cells, as well as testosterone production in the testes [29–35], possibly by disrupting the hypothalamus-pituitary-gonadal axis and/or via induction of oxidative stress [36–38]. Steroid hormone levels are important in all phases of fetal male reproductive system development, which begins at gestational week 6 with the differentiation of gonadal tissue into fetal testes and is mostly complete by week 15, although growth and testicular descent continue through the third trimester. Considering the complexity of this process throughout the gestational period, there is substantial potential for differential windows of susceptibility to exposure during this time. Indeed, both animal and human studies have demonstrated that exposure to androgens, anti-androgens, or estrogens during different periods of in utero development have different effects on the newborn male reproductive tract [13, 18, 19, 39, 40]. To our knowledge, the current study is the first to investigate the long term impact of phthalate and BPA exposure during specific periods of in utero development on male reproductive development during puberty.

Our analyses examining exposure levels and rates of change across pregnancy in relation to reproductive hormones and sexual maturation enabled us to look at exposure across time within the same mother/child pair rather than at discrete time points. Our findings using this method were consistent with those from our main analysis, thus our conclusions are robust to the method of analysis used.

Our analysis was limited by a relatively small sample size, and we made a large number of comparisons, increasing the likelihood of chance findings. Also, few boys had a PH Tanner stage >1, which may have resulted in unstable effect estimates (i.e. large confidence intervals). However, we are currently conducting an additional follow-up of over 550 children in the ELEMENT cohort, including boys in the current analysis who are now 9–17 years of age, for repeated hormone measurements and assessments of sexual maturation. Timing of the prenatal period may also have influenced our findings, as decreases in exposure to some phthalates have been demonstrated in the US since the early 2000s [41]. Because our study included a range of ages (8–14 years), older boys may have been more likely to have higher in utero phthalate exposure as well as to have begun puberty. However, it is not known if these same exposure trends occurred in Mexico during the time of mother’s enrollment in this study (1997–2004), and when we additionally adjusted our models for year of conception, our results did not materially change. We also measured serum hormones using standardized immunoassays rather than the preferred method of LC/MS-MS, although hormone concentrations were all above their respective LODs, with the exception of testosterone in 12 samples. In addition, hormone concentrations were measured at only one time point and not adjusted for time of day, leading to potential non-differential misclassification due to diurnal variation. Another limitation was measurements of urinary phthalate metabolites at only one time point during each trimester, which may not fully characterize exposure across in utero development.

## Conclusions

Our results suggest that associations between in utero phthalate and BPA exposure and peripubertal measures of male reproductive development are dependent on the timing of that exposure during gestation. These findings are consistent with current understanding of male reproductive tract development during the fetal and pubertal periods, and with reports from animal research. Although our findings need to be repeated in larger and more mature populations, future epidemiological studies of in utero exposure to endocrine disrupting chemicals and reproductive development should consider potential windows of susceptibility.

## Additional file

Additional file 1: Table S1. Spearman Correlations between trimester specific urinary specific gravity and phthalate metabolites or BPA measurements from that trimester. **Table S2.** Number of boys included in trimester-specific analyses who had Tanner Stage =1 vs > 1, and testicular volume ≤ 3 ml vs > 3 ml. **Table S3.** Odds of Tanner stage >1 or TV >3 ml associated with an IQR increase in uncorrected in utero urinary phthalate metabolite or BPA concentration. **Table S4.** Fixed and random effect estimates of gestational age on ln-transformed, SG-standardized phthalate metabolite and BPA concentrations in prenatal urine samples. (DOCX 29 kb)

## Abbreviations

AGD: Anogenital distance
BMI: Body mass index
BPA: Bisphenol A
CI: Confidence interval
DBP: Dibutyl phthalate
DEHP: Di-2-ethylhexyl phthalate
DHEA-S: Dehydroepiandrosterone sulfate
EDC: Endocrine disrupting chemical
ELEMENT: Early Life Exposure in Mexico to Environmental Toxicants
GD: Tanner genital stage
GM: Geometric mean
ICC: Intraclass correlation coefficient
ID-LC-MS/MS: Isotope dilution–liquid chromatography–tandem mass spectrometry
IQR: Interquartile range
LOD: Limit of detection
MBzP: Monobenzyl phthalate
MCPP: Mono-3-carboxypropyl phthalate
MECPP: Mono-2-ethyl-5-carboxypentyl phthalate
MEHHP: Mono-2-ethyl-5-hydroxyhexyl phthalate
MEHP: Mono-2-ethylhexyl phthalate
MEOHP: Mono-2-ethyl-5-oxohexyl phthalate
MEP: Monoethyl phthalate
MiBP: Monoisobutyl phthalate
MnBP: Mono-*n*-butyl phthalate
OR: Odds ratio
PH: Tanner pubic hair stage
SG: Specific gravity
SHBG: Sex hormone-binding globulin
TV: Testicular volume
