# Supplementary material for: Impact of phthalate and BPA exposure during in utero windows of susceptibility on reproductive hormones and sexual maturation in peripubertal males
Source: Environ Health. 2017 Jun 21;16:69. doi: 10.1186/s12940-017-0278-5 (PMC5480112; doi:10.1186/s12940-017-0278-5)
Supplement: Additional file 1: Table S1. — Spearman Correlations between trimester specific urinary specific gravity and phthalate metabolites or BPA measurements from that trimester. Table S2. Number of boys included in trimester-specific analyses who had Tanner Stage =1 vs > 1, and testicular volume ≤ 3 ml vs > 3 ml. Table S3. Odds of Tanner stage >1 or TV >3 ml associated with an IQR increase in uncorrected in utero urinary phthalate metabolite or BPA concentration. Table S4. Fixed and random effect estimates of gestational age on ln-transformed, SG-standardized phthalate metabolite and BPA concentrations in prenatal urine samples. (DOCX 29 kb) [file 12940_2017_278_MOESM1_ESM.docx]

**Impact of phthalate and BPA exposure during *in utero* windows of susceptibility on reproductive hormones and sexual maturation in peripubertal males**

Supplemental Material

Table of Contents:

| Table S1. Spearman Correlations between trimester specific urinary specific gravity and phthalate metabolites or BPA measurements from that trimester. | Page 2 |
| --- | --- |
| Table S2. Number of boys included in trimester-specific analyses who had Tanner Stage =1 vs > 1, and testicular volume ≤ 3ml vs > 3ml. | Page 3 |
| Table S3. Odds of Tanner stage >1 or TV >3ml associated with an IQR increase in uncorrected *in utero* urinary phthalate metabolite or BPA concentration. | Page 4 |
| Table S4. Fixed and random effect estimates of gestational age on ln-transformed, SG-standardized phthalate metabolite and BPA concentrations in prenatal urine samples. | Page 5 |

| Table S1. Spearman Correlations between trimester specific urinary specific gravity and phthalate metabolites or BPA measurements from that trimester. | | | |
| --- | --- | --- | --- |
|  | 1st trimester SG | 2nd trimester SG | 3rd trimester SG |
| 1st trimester SG | 1.00 | 0.18 | 0.29 |
| *p-value* |  | *0.01* | *<.0001* |
| 2nd trimester SG | 0.18 | 1.00 | 0.30 |
| *p-value* | *0.01* |  | *<.0001* |
| 3rd trimester SG |  | 0.30 | 1.00 |
| *p-value* |  | *<.0001* |  |
| BPA | 0.46 | 0.53 | 0.53 |
| *p-value* | *<.0001* | *<.0001* | *<.0001* |
| MEHP | 0.40 | 0.61 | 0.52 |
| *p-value* | *<.0001* | *<.0001* | *<.0001* |
| MEHHP | 0.45 | 0.64 | 0.60 |
| *p-value* | *<.0001* | *<.0001* | *<.0001* |
| MEOHP | 0.44 | 0.64 | 0.61 |
| *p-value* | *<.0001* | *<.0001* | *<.0001* |
| MECPP | 0.51 | 0.70 | 0.63 |
| *p-value* | *<.0001* | *<.0001* | *<.0001* |
| MBzP | 0.38 | 0.60 | 0.39 |
| *p-value* | *<.0001* | *<.0001* | *<.0001* |
| MBP | 0.46 | 0.60 | 0.56 |
| *p-value* | *<.0001* | *<.0001* | *<.0001* |
| MIBP | 0.40 | 0.60 | 0.52 |
| *p-value* | *<.0001* | *<.0001* | *<.0001* |
| MCPP | 0.52 | 0.72 | 0.59 |
| *p-value* | *<.0001* | *<.0001* | *<.0001* |
| MEP | 0.35 | 0.57 | 0.36 |
| *p-value* | *<.0001* | *<.0001* | *<.0001* |
| ΣDEHP | 0.48 | 0.67 | 0.63 |
| *p-value* | *<.0001* | *<.0001* | *<.0001* |
| ΣDBP | 0.47 | 0.60 | 0.57 |
| *p-value* | *<.0001* | *<.0001* | *<.0001* |

Table S2. Number of boys included in trimester-specific analyses who had Tanner Stage =1 vs > 1, and testicular volume ≤ 3ml vs > 3ml.

| Sexual Maturation Measure | | Visit 1 | | Visit 2 | | Visit 3 | | GM | |
| --- | --- | --- | --- | --- | --- | --- | --- | --- | --- |
|  |  | N (%) | | N (%) | | N (%) | | N (%) | |
| Genital Development | Tanner Stage >1 | 36 | (40) | 39 | (44) | 48 | (46) | 49 | (46) |
|  | Tanner Stage = 1 | 54 | (60) | 50 | (56) | 57 | (54) | 57 | (54) |
| Pubic Hair Development | Tanner Stage >1 | 9 | (10) | 12 | (13) | 15 | (14) | 16 | (15) |
|  | Tanner Stage = 1 | 81 | (90) | 77 | (87) | 90 | (86) | 90 | (85) |
| Testicular Volume | > 3ml | 73 | (82) | 73 | (83) | 87 | (84) | 88 | (84) |
|  | ≤ 3ml | 16 | (18) | 15 | (17) | 17 | (16) | 17 | (16) |

Table S3. Odds of Tanner stage >1 or TV >3ml associated with an IQR increase in uncorrected *in utero* urinary phthalate metabolite or BPA concentration. ^a^

|  | Visit 1 (n=90) | Visit 2 (n=89) | Visit 3 (n=105) | GM (n=106) |
| --- | --- | --- | --- | --- |
|  | OR (95%CI) | OR (95%CI) | OR (95%CI) | OR (95%CI) |
| Genital Development | |  |  |  |
| BPA | 0.69 (0.38, 1.26) | 1.71 (0.91, 3.21) | 0.91 (0.51, 1.61) | 1.06 (0.51, 2.2) |
| MBzP | 1 (0.62, 1.64) | 0.94 (0.54, 1.64) | 0.69 (0.36, 1.33) | 0.78 (0.39, 1.57) |
| MCPP | 1.3 (0.67, 2.51) | 1.14 (0.54, 2.4) | 0.99 (0.56, 1.77) | 1.23 (0.53, 2.84) |
| MEP | 1.49 (0.8, 2.8) | 1.21 (0.58, 2.52) | 1.09 (0.58, 2.04) | 1.29 (0.59, 2.8) |
| ΣDEHP | 1.02 (0.59, 1.76) | 0.85 (0.46, 1.57) | 0.75 (0.42, 1.34) | 0.85 (0.42, 1.74) |
| ΣDBP | 0.84 (0.51, 1.37) | 0.84 (0.47, 1.5) | 0.62 (0.32, 1.17) | 0.61 (0.29, 1.29) |
| PH Development | |  |  |  |
| BPA | 0.2 (0.01, 2.58) | 1.11 (0.32, 3.82) | 0.48 (0.14, 1.66) | 0.83 (0.22, 3.14) |
| MBzP | 0.07 (0, 1.83) | 1.12 (0.25, 5.08) | **0.17 (0.03, 0.89)*** | 0.32 (0.1, 1.02) |
| MCPP | 0.33 (0.02, 6.59) | 9.91 (0.63, 154.71) | 0.59 (0.21, 1.66) | 0.6 (0.14, 2.64) |
| MEP | 2.22 (0.35, 14.24) | 1.5 (0.22, 10.36) | 0.47 (0.16, 1.39) | 0.59 (0.18, 1.98) |
| ΣDEHP | 0.01 (<0.001, 2.2) | 3.15 (0.31, 32.26) | 0.35 (0.1, 1.19) | 0.18 (0.03, 1.2) |
| ΣDBP | 0.27 (0.01, 4.92) | 3.72 (0.51, 27.14) | 0.4 (0.13, 1.25) | 0.43 (0.12, 1.54) |
| Testicular Volume | |  |  |  |
| BPA | 1.25 (0.67, 2.34) | **2.59 (1.11, 6.08)*** | 1.25 (0.61, 2.55) | 2.5 (0.9, 6.93) |
| MBzP | 0.72 (0.41, 1.27) | 0.71 (0.35, 1.44) | 0.69 (0.3, 1.59) | 0.45 (0.18, 1.14) |
| MCPP | 0.63 (0.29, 1.36) | 2.01 (0.77, 5.28) | 1.09 (0.53, 2.27) | 1.29 (0.45, 3.7) |
| MEP | 1.32 (0.64, 2.7) | 0.88 (0.37, 2.12) | 1.37 (0.6, 3.11) | 1.23 (0.47, 3.26) |
| ΣDEHP | 1.2 (0.66, 2.17) | 1.18 (0.58, 2.4) | 1.71 (0.85, 3.46) | 2.22 (0.83, 5.9) |
| ΣDBP | 1.04 (0.57, 1.89) | 1.92 (0.93, 3.98) | 1.02 (0.5, 2.12) | 1.62 (0.62, 4.25) |

*p<0.05; ^a^ Adjusted for child age, BMI z-score, and urinary specific gravity.

Table S4. Fixed and random effect estimates of gestational age on ln-transformed, SG-standardized phthalate metabolite and BPA concentrations in prenatal urine samples.

|  | Population Mean  (fixed effect)^a^ | Distribution of Random Effects of Gestational Age on Prenatal Urinary Phthalate and BPA Concentrations | | | | | | | | |
| --- | --- | --- | --- | --- | --- | --- | --- | --- | --- | --- |
|  |  | Mean^b^ | SD | Min | 5^th^ | 25^th^ | 50^th^ | 75^th^ | 95^th^ | Max |
| All mothers (n=224) | |  |  |  |  |  |  |  |  |  |
| intercepts |  |  |  |  |  |  |  |  |  |  |
| BPA | 0.23 | 0 | 0.524 | -1.695 | -0.771 | -0.346 | -0.041 | 0.326 | 0.875 | 1.824 |
| MBzP | 0.79 | 0 | 0.774 | -2.276 | -1.240 | -0.507 | -0.024 | 0.499 | 1.309 | 2.427 |
| MCPP | 0.14 | 0 | 0.515 | -1.363 | -0.798 | -0.329 | 0.005 | 0.267 | 0.794 | 2.393 |
| MEP | 5.06 | 0 | 0.769 | -1.942 | -1.215 | -0.588 | 0.010 | 0.465 | 1.342 | 2.087 |
| DEHP | -1.55 | 0 | 0.232 | -0.629 | -0.430 | -0.136 | -0.005 | 0.152 | 0.375 | 0.622 |
| DBP | -1.26 | 0 | 0.604 | -1.542 | -0.901 | -0.440 | -0.002 | 0.389 | 1.065 | 2.317 |
| slopes |  |  |  |  |  |  |  |  |  |  |
| BPA | -0.012 | 0 | 0.012 | -0.043 | -0.020 | -0.007 | 0.000 | 0.007 | 0.019 | 0.038 |
| MBzP | 0.027 | 0 | 0.021 | -0.066 | -0.035 | -0.014 | 0.000 | 0.013 | 0.033 | 0.056 |
| MCPP | 0.006 | 0 | 0.018 | -0.075 | -0.026 | -0.011 | 0.000 | 0.011 | 0.029 | 0.060 |
| MEP | -0.004 | 0 | 0.017 | -0.048 | -0.030 | -0.009 | 0.000 | 0.010 | 0.030 | 0.048 |
| DEHP | 0.012 | 0 | 0.006 | -0.017 | -0.009 | -0.004 | 0.000 | 0.004 | 0.009 | 0.019 |
| DBP | 0.003 | 0 | 0.016 | -0.053 | -0.025 | -0.010 | -0.001 | 0.009 | 0.026 | 0.047 |
| Mothers who gave birth to boys (n=105) | | | |  |  |  |  |  |  |  |
| intercepts |  |  |  |  |  |  |  |  |  |  |
| BPA |  | -0.088 | 0.493 | -1.695 | -0.865 | -0.399 | -0.087 | 0.203 | 0.668 | 1.416 |
| MBzP |  | -0.013 | 0.762 | -2.276 | -1.246 | -0.428 | -0.024 | 0.406 | 1.261 | 1.569 |
| MCPP |  | -0.009 | 0.444 | -1.204 | -0.723 | -0.287 | -0.002 | 0.252 | 0.756 | 1.154 |
| MEP |  | -0.045 | 0.752 | -1.942 | -1.233 | -0.588 | -0.063 | 0.415 | 1.286 | 2.087 |
| ΣDEHP |  | -0.023 | 0.228 | -0.629 | -0.434 | -0.141 | -0.022 | 0.134 | 0.319 | 0.494 |
| ΣDBP |  | -0.065 | 0.590 | -1.542 | -0.955 | -0.448 | -0.111 | 0.320 | 1.065 | 1.320 |
| slopes |  |  |  |  |  |  |  |  |  |  |
| BPA |  | 0.0004 | 0.012 | -0.029 | -0.019 | -0.007 | 0.000 | 0.007 | 0.019 | 0.038 |
| MBzP |  | 0.0003 | 0.021 | -0.046 | -0.035 | -0.011 | 0.001 | 0.012 | 0.035 | 0.054 |
| MCPP |  | 0.0000 | 0.017 | -0.043 | -0.026 | -0.011 | -0.001 | 0.010 | 0.026 | 0.060 |
| MEP |  | 0.0002 | 0.015 | -0.042 | -0.021 | -0.009 | -0.001 | 0.008 | 0.029 | 0.045 |
| ΣDEHP |  | 0.0001 | 0.006 | -0.017 | -0.008 | -0.004 | 0.000 | 0.003 | 0.009 | 0.015 |
| ΣDBP |  | 0.001 | 0.015 | -0.035 | -0.022 | -0.010 | -0.001 | 0.010 | 0.024 | 0.047 |

^a^ Population fixed effects based on models including mothers who gave birth to both male and female infants (n=224).

^b^ Random effect means are based on mothers who gave birth to male infants and are included in the present analyses (n=105). The random effect mean represents the average deviation from the overall fixed effect mean in this subset of the population.

^c^ Intercepts represent urinary phthalate metabolite or BPA concentrations at 7 weeks gestation.

^d^ Slopes represent the rate of change in urinary phthalate metabolite or BPA concentrations across pregnancy.
